# Etiological Spectrum of Hypercalcemia in a North Indian Tertiary Care Center: Emerging Role of Vitamin D Toxicity

**DOI:** 10.7759/cureus.108257

**Published:** 2026-05-04

**Authors:** Jibran A Sheikh, Zhahid Baigh, Baleekhudin Mohd O Dawar

**Affiliations:** 1 Internal Medicine, Government Medical College Srinagar, Srinagar, IND; 2 Internal Medicine, Government Medical College Jammu, Jammu, IND

**Keywords:** hypercalcemia, iatrogenic disease, india, vitamin d deficiency, vitamin d toxicity

## Abstract

Introduction: Globally, primary hyperparathyroidism and malignancy account for the majority of hypercalcemia cases. However, regional epidemiological variations exist. Kashmir, a high-altitude region in North India with a high prevalence of vitamin D deficiency, has witnessed increasing use of high-dose vitamin D supplementation. This has led to growing concern about iatrogenic hypercalcemia secondary to hypervitaminosis D. This study aimed to evaluate the current etiological spectrum of hypercalcemia among hospitalized patients in Kashmir and to assess the contribution of vitamin D toxicity and its underlying causes. Materials and methods: This retrospective study included adult patients (≥18 years) admitted to a tertiary care center in Kashmir between 1 January 2025 and 30 June 2025 with corrected serum calcium >10.5 mg/dL on at least two occasions. Demographic data, biochemical parameters, and etiological diagnoses were retrieved from hospital records. Data were analyzed using descriptive statistics, and comparisons between groups were performed using the Student’s t-test, Mann-Whitney U test, and chi-square test. Results: A total of 40 patients were included (mean age 62 ± 12.8 years; 52.5% female). Malignancy (including multiple myeloma and lymphoma) was the most common cause of hypercalcemia, followed by primary hyperparathyroidism (20%) and vitamin D toxicity (20%). Patients with hypervitaminosis D had markedly elevated 25-hydroxyvitamin D levels (123.5 ± 25 ng/mL) with suppressed intact parathyroid hormone (iPTH) levels. Patients with malignancy-associated hypercalcemia were significantly older than those with non-malignant causes, whereas serum 25-hydroxyvitamin D levels and iPTH were significantly higher in the non-malignant group. Review of prior regional studies demonstrated frequent administration of high-dose intramuscular cholecalciferol (600,000 IU) and repeated high-dose oral supplementation as major contributors to toxicity. Conclusions: While malignancy and primary hyperparathyroidism remain leading causes of hypercalcemia in hospitalized patients, vitamin D toxicity now represents a significant and preventable contributor in the Kashmiri population. Greater emphasis on rational prescribing, avoidance of high-dose intermittent regimens, and appropriate biochemical monitoring is warranted to reduce iatrogenic hypercalcemia.

## Introduction

Hypercalcemia is a common metabolic abnormality encountered in hospitalized patients. Globally, over 90% of cases are attributed to primary hyperparathyroidism and malignancy [1]. However, the etiological spectrum varies across populations depending on demographics, environmental factors, and prescribing practices.

Kashmir, a high-altitude region in North India, has a high reported prevalence of vitamin D deficiency [2-4]. This has resulted in widespread supplementation practices, often involving high-dose intermittent regimens, including intramuscular (IM) cholecalciferol (600,000 IU) and repeated high-dose oral formulations. While vitamin D replacement is essential in deficiency states, excessive or unsupervised administration may result in hypervitaminosis D and subsequent hypercalcemia.

Recent regional reports suggest a growing contribution of vitamin D toxicity to hypercalcemia cases [5-8]. However, contemporary data on the etiological distribution of hypercalcemia in hospitalized patients in this region, along with analyses of the factors contributing to vitamin D toxicity, remain limited.

This study aimed to characterize the etiological spectrum of hypercalcemia in hospitalized patients over a six-month period, quantify the contribution of vitamin D toxicity, and contextualize the findings in relation to existing literature from North India.

## Materials and methods

This retrospective observational study was conducted at Shri Maharaja Hari Singh Hospital, Srinagar, Jammu and Kashmir, India. Data for all patients admitted to Medical Unit 6 of the hospital with a diagnosis of hypercalcemia between 1 January 2025 and 30 June 2025 were retrieved from the hospital medical records. Adults ≥18 years of age with corrected serum calcium >10.5 mg/dL on at least two different occasions were included. Serum calcium was corrected for albumin using the formula: corrected calcium (mg/dL) = measured serum calcium + 0.8 × (4.0 − serum albumin (g/dL)).

Data on age, sex, serum calcium, intact parathyroid hormone (iPTH), 25-hydroxyvitamin D levels, and final etiological diagnosis, as documented in the chart, were collected. Patients with incomplete records were excluded from the study. Vitamin D toxicity was defined as a 25-hydroxyvitamin D level >100 ng/mL, suppressed iPTH, and a compatible clinical context [9].

Statistical analysis was conducted using SPSS Statistics version 28.0.1.1 (IBM Corp. Released 2022. IBM SPSS Statistics for Mac, Version 28.0.1.1. Armonk, NY: IBM Corp.). Descriptive statistics were expressed as mean ± standard deviation for continuous variables and as percentages for categorical variables. Normality of continuous variables was assessed using the Shapiro-Wilk test. Normally distributed variables were compared using the Student’s t-test, while non-normally distributed variables were compared using the Mann-Whitney U test. Categorical variables were compared using the chi-square test. A p-value <0.05 was considered statistically significant.

## Results

A total of 671 patients were screened. Forty-two patients met the inclusion criteria; however, two were excluded due to incomplete records. The final analysis included 40 patients. Baseline characteristics of the cohort are provided in Table 1.

**Table 1:** Baseline characteristics Data presented as mean ± standard deviation or percentage. iPTH: intact parathyroid hormone

Variable	Value	Reference range
Age	62 ± 12.8 years	-
Serum calcium	12.7 ± 1.9 mg/dL	8.6-10.2 mg/dL
Serum phosphorus	3.7 ± 1.3 mg/dL	3-4.5 mg/dL
Serum iPTH	143 ± 359 pg/mL	10-65 pg/mL
Serum 25-hydroxyvitamin D	53 ± 44 ng/mL	30-60 ng/mL
Male	47.50%	-
Female	52.50%	-

Table 2 presents the etiological breakdown of hypercalcemia in the study. Among malignancy-associated cases, multiple myeloma and lymphoma were the most common.

**Table 2:** Etiology of hypercalcemia

Diagnosis	Number of patients	Percentage of patients
Multiple myeloma	13	32%
Primary hyperparathyroidism	8	20%
Vitamin D toxicity	8	20%
Lymphoma	3	7.50%
Milk alkali syndrome	3	7.50%
Sarcoidosis	2	5%
Squamous cell carcinoma of the lung	1	2.50%
Tertiary hyperparathyroidism	1	2.50%
Parathyroid carcinoma	1	2.50%

A comparison of clinical and biochemical parameters between patients with vitamin D toxicity and those with other causes of hypercalcemia is provided in Table 3.

**Table 3:** Comparison of patients with vitamin D toxicity and other etiologies of hypercalcemia Data presented as mean ± standard deviation or percentage. iPTH: intact parathyroid hormone

Variable	Vitamin D toxicity (n = 8)	Other causes (n = 32)	Test statistic	p-value
Age (years)	64.6 ± 9.8	62.16 ± 13.7	t = 0.50	0.62
Serum calcium (mg/dL)	12.57 ± 2	12.73 ± 1.96	U = 121	0.81
Serum phosphorus (mg/dL)	3.5 ± 0.73	3.77 ± 1.39	U = 116	0.71
Serum iPTH (pg/mL)	21.2 ± 12.2	174.3 ± 396.6	U = 100.5	0.35
Serum 25-hydroxyvitamin D (ng/mL)	123.5 ± 25	36.33 ± 27.2	U = 5	<0.001
Male	75%	59%	χ² = 0.71	0.4

Serum 25-hydroxyvitamin D levels were significantly higher in the vitamin D toxicity group (123.5 ± 25 ng/mL vs 36.33 ± 27.2 ng/mL, p < 0.001). There were no statistically significant differences between the two groups with respect to age (64.6 ± 9.8 vs 62.16 ± 13.7 years, p = 0.62), serum calcium (12.57 ± 2 vs 12.73 ± 1.96 mg/dL, p = 0.81), serum phosphorus (3.5 ± 0.73 vs 3.77 ± 1.39 mg/dL, p = 0.71), or sex distribution (p = 0.40). Although iPTH levels were lower in the vitamin D toxicity group (21.2 ± 12.2 vs 174.3 ± 396.6 pg/mL), this difference did not reach statistical significance (p = 0.35). A comparison between patients with malignancy-associated and non-malignant causes of hypercalcemia is shown in Table 4.

**Table 4:** Comparison of clinical and biochemical parameters between malignant and non-malignant causes of hypercalcemia Data presented as mean ± standard deviation or percentage. iPTH: intact parathyroid hormone

Variable	Malignant (n = 18)	Non-malignant (n = 22)	Test statistic	p-value
Age (years)	69.17 ± 12.45	57.32 ± 10.86	t = 3.12	0.003
Serum calcium (mg/dL)	12.91 ± 2.02	12.53 ± 1.91	U = 181	0.55
Serum phosphorus (mg/dL)	3.96 ± 1.37	3.52 ± 1.2	U = 152	0.18
Serum iPTH (pg/mL)	72.72 ± 232.6	201.7 ± 433.3	U = 110	0.03
Serum 25-hydroxyvitamin D (ng/mL)	31.23 ± 16.23	72.2 ± 51.1	U = 74	0.002
Male	56%	59%	χ² = 0.04	0.84

Patients with malignancy were significantly older than those with non-malignant etiologies (69.17 ± 12.45 vs 57.32 ± 10.86 years, p = 0.003). Serum 25-hydroxyvitamin D and iPTH levels were significantly higher in the non-malignant group (72.2 ± 51.1 vs 31.23 ± 16.23 ng/mL, p = 0.002, and 201.7 ± 433.3 vs 72.72 ± 232.6 pg/mL, p = 0.03, respectively). There were no statistically significant differences between the two groups with respect to serum calcium (p = 0.55), serum phosphorus (p = 0.18), or sex distribution (p = 0.84).

## Discussion

In our study, malignancy and primary hyperparathyroidism were the leading causes of hypercalcemia among hospitalized patients. This is consistent with published literature from tertiary care centers globally and within South Asia.

However, vitamin D-related hypercalcemia accounted for 20% of the total cases. As expected, vitamin D levels in this cohort were significantly higher, with suppressed iPTH, and most patients had moderate hypercalcemia. In our study, serum 25-hydroxyvitamin D and iPTH levels were significantly lower in patients with malignancy-associated hypercalcemia than in those with non-malignant causes. This finding likely reflects the presence of vitamin D toxicity cases within the non-malignant group as well as the increased prevalence of vitamin D deficiency or insufficiency in the cancer population due to poor nutrition, cachexia, and reduced sunlight exposure.

Our findings are consistent with pooled data from four previous regional studies conducted in Kashmir, in which vitamin D toxicity accounted for 46 of 259 hypercalcemia cases (17.8%) [5-8]. Notably, the first of these studies was published in 2016. Despite early recognition of the magnitude of the problem, the prevalence of vitamin D toxicity appears to have remained largely unchanged over the subsequent decade.

As discussed above, prior studies have documented a high prevalence of vitamin D deficiency in the Kashmiri population. The widespread deficiency is multifactorial, attributed to geographic latitude, limited sun exposure related to clothing practices, and the absence of routine food fortification [2]. Since recognition of this public health concern, vitamin D supplementation, both prescribed and over-the-counter, has become widespread. While supplementation is often appropriate, its liberal and sometimes unsupervised use has contributed to an increasing incidence of vitamin D toxicity. Correctable factors contributing to this trend are outlined below:

Repeated administration of high-dose vitamin D (megadoses)

Nearly all regional studies examining vitamin D toxicity have identified repeated administration of 600,000 international units (IU) intramuscular (IM) cholecalciferol as the principal contributor, with comparatively fewer cases attributable to frequent or prolonged use of 60,000 IU oral formulations. Table 5 lists four such studies from the region.

**Table 5:** Studies of vitamin D toxicity from North India with vitamin D formulation and cumulative dosing data IM: intramuscular, IU: international units, NA: not available

Study	Location	Number of patients	Vitamin D formulation used	Cumulative vitamin D dose
Misgar et al. 2019 [10]	Kashmir, India	32	Multiple 600,000 IU IM injections	1,800,000-30,000,000 IU
Kaur et al. 2015 [11]	Delhi, India	16	Multiple 600,000 IU IM injections and/or multiple 60,000 IU sachets	2,220,000-60,000,000 IU
Sath et al. 2016 [12]	Kashmir, India	11	Multiple 600,000 IU IM injections and/or multiple 60,000 IU sachets	NA
Koul et al. 2011 [13]	Kashmir, India	10	Multiple 600,000 IU IM injections and/or multiple 60,000 IU sachets	3,600,000 IU-210,000,000 IU

Notably, the cumulative vitamin D doses reported in these studies ranged from 1.8 to 210 million IU, typically administered over weeks to a few months. The 600,000 IU IM formulation is not routinely used in most countries, including the United States and European nations. Limited safety data suggest that it can be administered safely as a single annual dose [14,15]. Previous studies with annual IM injections of 300,000 IU did not show any benefit in fracture reduction and suggested a possible increase in fracture risk. Studies have also shown an increase in bone turnover after such high doses [16]. For these reasons and concerns about toxicity without additional benefit, most professional societies recommend daily low-dose supplementation rather than intermittent high-dose supplementation [17].

Failure to monitor serum 25-hydroxyvitamin D and calcium levels after high-dose supplementation

Routine monitoring of vitamin D levels is not recommended for individuals taking vitamin D doses within the recommended daily intake [17]. However, individuals receiving higher-than-recommended doses for specific indications, such as malabsorption syndromes or to improve adherence, should undergo periodic monitoring of serum 25-hydroxyvitamin D levels to prevent overcorrection and toxicity. In the largest study of hypervitaminosis D from Kashmir, none of the 32 participants had their vitamin D levels monitored before or during therapy [10].

Limited regulatory oversight of high-dose vitamin D formulations

Vitamin D, even in doses ≥60,000 IU, is available over the counter in India. In the study by Misgar et al., 10% of patients with vitamin D toxicity were taking megadoses of vitamin D without a prescription [10].

Inappropriate prescribing of vitamin D in patients without established indications

The various indications for which most patients had received vitamin D in two of the four listed studies included non-specific aches, osteoarthritis, fatigue, and back pain [10,13].

In view of the above, current prescribing and monitoring practices for vitamin D require revision. Lower-dose daily oral supplementation should be preferred over high-dose intermittent therapy [17]. In the rare instances where high-dose therapy is warranted, regular monitoring of vitamin D levels should be undertaken. Vitamin D supplementation should only be provided to specific populations where the benefit of its use has been demonstrated or is likely (osteomalacia, osteoporosis, hypocalcemia, hyperparathyroidism, pregnancy, high-risk prediabetes, and older age) and should strictly be avoided in conditions where it provides no benefit (osteoarthritis, non-specific pains, fatigue, and spondylosis) [17]. Routine testing and replacement in an otherwise healthy population are also not recommended due to the lack of any demonstrable benefit [17]. There also has to be stricter regulation of higher-dose vitamin D available over the counter, especially the 600,000 IU IM formulation and the 60,000 IU oral formulation. Only formulations within the safe daily intake limit should be sold over the counter. All of this must be done in parallel with public education campaigns about the risks of vitamin D supplementation and its limited benefits, which apply only to appropriately selected individuals.

Limitations

Limitations of our study include its single-center, small-sample, retrospective design. The relatively small sample size and single-center design may have limited the statistical power to detect significant differences between some clinical and biochemical parameters across etiological subgroups. The study was limited to univariate analysis, and multivariate analysis was not performed due to the relatively small sample size, which may limit the ability to adjust for potential confounders. Although detailed data on the dose and duration of vitamin D supplementation were unavailable, this limitation was partially addressed by interpreting our findings in light of previously published regional studies reporting these parameters. However, the study also has important strengths. It provides contemporary, real-world data from a region with high rates of vitamin D supplementation and contextualizes the findings through a pooled regional literature review. To our knowledge, few recent analyses have examined the evolving etiological spectrum of hypercalcemia in this population.

## Conclusions

Malignancy and primary hyperparathyroidism remain the leading causes of hypercalcemia among hospitalized patients in Kashmir. However, vitamin D toxicity constitutes a significant and preventable proportion of cases. Despite recognition of widespread vitamin D deficiency in the region, the burden of hypervitaminosis D-related hypercalcemia has not declined over the past decade. Rational prescribing practices, avoidance of high-dose intermittent regimens in routine deficiency states, and appropriate biochemical monitoring are essential to reduce iatrogenic hypercalcemia.
